# Lifestyle Acquired Immunity, Decentralized Intelligent Infrastructures, and Revised Healthcare Expenditures May Limit Pandemic Catastrophe: A Lesson From COVID-19

**DOI:** 10.3389/fpubh.2020.566114

**Published:** 2020-11-05

**Authors:** Asif Ahmed, Tasnima Haque, Mohammad Mahmudur Rahman

**Affiliations:** ^1^Biotechnology and Genetic Engineering Discipline, Khulna University, Khulna, Bangladesh; ^2^Bangladesh Institute of Health Sciences General Hospital, Dhaka, Bangladesh; ^3^Department of Medical Biotechnology, Bangladesh University of Health Sciences, Dhaka, Bangladesh

**Keywords:** COVID-19, socioeconomic, demographic, health indicator, urban population, median age

## Abstract

Throughout history, the human race has often faced pandemics with substantial numbers of fatalities. As the COVID-19 pandemic has now affected the whole planet, even countries with moderate to strong healthcare support and expenditure have struggled to contain disease transmission and casualties. Countries affected by COVID-19 have different demographics, socioeconomic, and lifestyle health indicators. In this context, it is important to find out to what extent these parametric variations are modulating disease outcomes. To answer this, this study selected demographic, socioeconomic, and health indicators e.g., population density, percentage of the urban population, median age, health expenditure per capita, obesity, diabetes prevalence, alcohol intake, tobacco use, case fatality of non-communicable diseases (NCDs) as independent variables. Countries were grouped according to these variables and influence on dependent variables e.g., COVID-19 positive tests, case fatality, and case recovery rates were statistically analyzed. The results suggested that countries with variable median age had a significantly different outcome on positive test rate (*P* < 0.01). Both the median age (*P* = 0.0397) and health expenditure per capita (*P* = 0.0041) showed a positive relation with case recovery. An increasing number of tests per 100 K of the population showed a positive and negative relationship with the number of positives per 100 K population (*P* = 0.0001) and the percentage of positive tests (*P* < 0.0001), respectively. Alcohol intake per capita in liter (*P* = 0.0046), diabetes prevalence (*P* = 0.0389), and NCDs mortalities (*P* = 0.0477) also showed a statistical relation to the case fatality rate. Further analysis revealed that countries with high healthcare expenditure along with high median age and increased urban population showed more case fatality but also had a better recovery rate. Investment in the health sector alone is insufficient in controlling the severity of the pandemic. Intelligent and sustainable healthcare both in urban and rural settings and healthy lifestyle acquired immunity may reduce disease transmission and comorbidity induced fatalities, respectively.

## Introduction

There have been rapid demographic changes in most regions and countries of the world since the middle of the last century. Increased population density, urban population, and life expectancy are noticeable examples of such changes (1, 2). The key objective of these transitions is connected to improving the socioeconomic level of a country's population. Socioeconomic development influences population health status through the regulation of the environment, lifestyle, and healthcare systems (3). Socioeconomic variables including population density, gross national income (GNI) per capita, and health expenditure per capita play an important role in achieving sustainable development (4, 5). The health policies of governments are important and perhaps the most critical aspect of these in ensuring adequate facilities and management in the population (6).

Over the last few decades, many governments have increased spending in health sectors to improve healthcare systems, treatments, research, and the development of new drugs and vaccines, and technologies for preventing and controlling diseases (7–12). The majority of this funding is being invested to prevent and treat diseases of a communicable and non-communicable nature. The amount spent on health is mostly dependent on GNI at purchasing power parity (PPP) (5, 13, 14) and the outcome is visible as reduced child mortality, increase in median age, and life expectancy at birth (15–18). Therefore, life expectancy, median age, and the percentage of the urban population have become the key indicators in human development indexes (19–21). The top-listed economies by GNI (PPP) are conventionally ahead in technology, research, and training (22).

Chronic respiratory diseases are responsible for almost four million premature deaths globally (23). Respiratory infections are usually worsened through population density, human behavior, insufficient public health safety, the genomic mutations of microbes, extraneous usage, and developing resistance to antibiotics. The lack of global coordination to prevent infectious disease outbreak and pandemic is partly due to weak policies, management, and expenditures in autocratic regimes, putting global health security at risk (24). In addition, developed countries have created facilities, readiness, and prevention from several life-threatening diseases without any divergences in rural and urban populations (24). However, these sustainably developed countries are facing devastating disasters during the Corona Virus Disease-19 (COVID-19) pandemic (25). COVID-19 emerged in December 2019 in Wuhan, China, with growing morbidity and mortality worldwide (26). As of May 22, 2020, there were more than 5.1 million positive cases and more than 0.3 million deaths (27).

The COVID-19 pandemic is caused by a positive-sense single stranded RNA (+ssRNA) virus named SARS-CoV-2 which belongs to the corona virus family. This family of viruses is capable of introducing human sickness (26) with an incubation period ranging from 2 to 14 days to develop symptoms (28). SARS-CoV-2 is mostly transmitted between persons via respiratory droplets, coughs, sneezes, and fomites (2). COVID-19 patients could be asymptomatic or develop flu-like symptoms with fever, dry cough, tiredness, and shortness of breath. Intensive care with ventilation and symptom-based therapies are needed for critical patients (2). The World Health Organization (WHO) declared the COVID-19 outbreak as a Public Health Emergency of International Concern (PHEIC) on January 30, a and pandemic on March 11, 2020 (29).

Clinical reports have confirmed that non-communicable disease (NCDs) including diabetes, heart disease, hypertension, respiratory disease (COPD/bronchial asthma), cancer, predominantly amongst the aged individuals, upsurge the susceptibility to COVID-19 (30). Surprisingly, countries with developed facilities to manage NCDs are struggling in the COVID-19 pandemic. Healthcare personnel are also being infected in all countries regardless of the country's economic status and demographic characters (24, 31). Several disease susceptibility patterns and predictions are also made to understand trends of infections, death, and recovery patterns (6, 32) and factors that influence transmission and fatalities (6). The associations of different environmental factors have also been investigated (6, 33, 34). COVID-19 requires sufficient public attention and needs to reprioritize financial involvement in appropriate segments of the health sector to confirm inclusive responses. Highly affected countries have used numerous tactics of financial distribution, depending on their capabilities, structures, and regulatory systems (35). In-depth investigations need to be conducted on the association of infection pattern, fatalities, and recovery in combination with population density, median age, percentage of the urban population, GDP per capita, and health expenditure per capita along with lifestyle and health indicators.

This study used publicly available demographic, socioeconomic, and health indicator data from COVID-19 affected countries to analyze and extrapolate the influence of categorical independent variables on disease outcome. The main research question asked to what degree these variables have a significant impact on the dependent variables including positive test rates, case fatality rates, and case recovery rates. These findings along with other studies of a similar nature might help to strengthen our preparedness to face any yet-to-come contagious pandemics in the near future. The findings of this study also highlight the importance of implementing an intelligent healthcare system in both urban and rural areas, coupled with a healthy lifestyle that boosts population immunity. Refocusing healthcare investment in poorly addressed sectors should be prioritized to minimize loss of life and related economic losses on a global scale.

## Methods

### Data Characteristics

The study was designed to use available secondary data for all variables. We obtained data on COVID-19 data of total positive cases, total death cases, total recovered cases, and total tests from Worldometers.info (27). The socioeconomic and demographic data, including total population, population density, median age, urban population percentages, male to female ratio, and financial information together with gross domestic product (GDP) in USD, gross national income (GNI) per capita (purchasing power parity, PPP) in USD, health expenditure (% of GDP) in USD were attained from the databank of the World Bank (36). However, the health expenditure per capita data of Hong Kong was obtained from the website of the Department of Health of the Government of the Hong Kong Special Administrative Region (37). In addition, GNI per capita (PPP) of Djibouti was obtained from the database of the International Monetary Fund (38). Lifestyle and health indicator data including the prevalence of obesity (39), the prevalence of insufficient physical activity (40), the prevalence of diabetes (41), NCD mortality rate per 100 K population (42), alcohol consumption per capita per liter per year (43), and tobacco use percentages of males (44) were obtained from the WHO and World Bank.

### Inclusion and Exclusion Criteria

In total, 91 countries were selected, whose total infection cases were over 1,000 on May 9, 2020 (Supplementary Data Sheet 1). For some particular parameters, the data relating to some countries were unavailable and they were excluded from the relevant analysis. However, other data from these countries were used in the respective analysis.

### Variables

The current study was conducted with several variables. Variables related to COVID-19, viz. total positive cases, total death cases, total recovered cases, the total number of tests were used. The percentage of positive tests were calculated against total number of tests performed. Case fatality and case recovered rates were calculated against total positive cases. The percentage of positive tests, case fatality, and case recovered rates were used as dependent variables instead of total positive cases, death cases, and recovery cases. This was because the socioeconomic conditions in a number of countries did not go through strategies of mass diagnosis, These countries instead conducted diagnosis when COVID-19 related symptoms first appeared. Population density, median age, percentage of the urban population, GNI per capita (PPP) in USD, and health expenditure per capita in USD were used as socioeconomic and demographic independent variables. Lifestyle and health related independent variables viz. prevalence of obesity, insufficient physical activity, diabetes, and any kind of tobacco use by men and women were obtained as percentages. Alcohol consumption per capita per liter was used as a variable, and the average number of liters consumed per year was considered. In addition, NCDs and mortality rates per 100 K of the population were also taken as another independent variable. Test numbers of countries were not uniform and depend on several factors including socioeconomic status and government initiatives. We calculated the number of tests per 100 K populations as well as positive cases per 100 K populations. Tests per 100 K of the population were used as independent variables and analyzed against positive test rates, case fatalities, and case recovered rate along with positive cases per 100 k as dependent variables. Linear regression analysis was performed with ungrouped independent variables with dependent variables as one to one analysis fashion. Later, the group wise distribution of independent variables were analyzed with dependent variables to find associations.

### Independent Variables Processing and Grouping

Independent variables except for GNI per capita (PPP) were grouped for trends and scenarios concerning dependent variables (Table 1). The GNI per capita (PPP) in USD were grouped into categories according to the World Bank (45). Grouped independent variables were analyzed against dependent variables as mean ± SEM. Cross analysis of each independent variable was also performed to get in-depth information, as outlined in the discussion section of this study. Cross analysis derived inter relational table can be found in Supplementary Table 2.

**Table 1:** Characteristics of grouped independent variables.

**Variables**	**Groups** **description**	**Numbers of** **country**	**Unavailable data** **(no of country)**
Population density Km^2^	Up to 100	51 (56.04%)	0
	100+ to 250	26 (28.57%)	
	250+ to 500	8 (8.79%)	
	500+	6 (6.59%)	
Per capita GNI (PPP) in USD^*^	Up to 1026	0	0
	1,026+ to 3,995	4 (3.40%)	
	3,995+ to 12,375	19 (20.88%)	
	12,375+	68 (74.73%)	
Urban population, %	Up to 50%	12 (13.19%)	0
	50+ to 75%	38 (41.76%)	
	75%+	41 (45.05%)	
Median age, years	Up to 20	6 (6.59%)	0
	20+ to 30	21 (23.08%)	
	30+ to 40	30 (32.97%)	
	40+	34 (37.36%)	
Per capita health expenditure in USD	Up to 100	12 (13.19%)	0
	100+ to 500	28 (30.77%)	
	500+ to 2,000	26 (28.57%)	
	2,000+	25 (27.47%)	
Prevalence of obesity, %	Up to 15%	19 (20.88%)	3 (3.30%)
	15+ to 20%	41 (45.05%)	
	20%+	28 (30.77%)	
Prevalence of insufficient physical activities, %	Below 20%	9 (9.89%)	
	20 to 30%	22 (24.18%)	14 (15.38%)
	30+ to 40%	35 (38.46%)	
	40%+	11 (12.09%)	
NCD mortality per 100 K	Up to 300	7 (7.69%)	5 (5.49%)
	300+ to 400	30 (32.97%)	
	400+ to 500	22 (24.18%)	
	500+ to 600	14 (15.38%)	
	600+ to 700	11 (12.09%)	
	700+	12 (13.19%)	
Prevalence of diabetes, %	Up to 5%	16 (17.58%)	1 (1.10%)
	5+ to 10%	56 (61.54%)	
	10 to 15%	8 (8.79%)	
	15%+	8 (8.79%)	
Alcohol consumptions per capita in liters per year	Up to 5 L	32 (35.16%)	2 (2.20%)
	5+ to 10 L	35 (38.46%)	
	10+	16 (17.58%)	
Any kind of tobacco used by male, % of population	Up to 25%	24 (26.37%)	2 (2.20%)
	25 to 40%	29 (31.87%)	
	40%+	19 (20.88%)	

*Groups were formed according to the distribution*.

* *Grouping of Per capita GNI (PPP) was done according to the World Bank classification by income level*.

### Statistical Analysis

We performed one to one regression analysis of ungrouped data in SPSS version 26. GraphPad Prism version 6 was used to generate graphs. One- or two- way ANOVA were performed as required along with the individual group to group statistical variation analysis. All statistical significance was measured at a significant value <0.05. All the final graphs generated in GraphPad Prism were combined using Inkscape version 0.92 graphics software.

## Results

The COVID-19 pandemic shows uneven epidemiological and clinical trends as it spreads to countries with climatic, socio-economic, lifestyle and demographic variations around the globe. In addition to mutation induced genomic variations in the virus, these factors might have a substantial influence on key outcomes like rate of infection, case fatality, and case recovery. This study was conducted to find possible links between the rate of COVID-19 infections, fatalities, and recovery with socioeconomic, demographic, lifestyles, and health indicators.

The association of dependent variables with non-grouped independent variables were measured with linear regression and results were shown in Table 2. One to one regression analysis showed that median age (*P* = 0.005), and any kind of tobacco use by men (*P* = 0.004) and women (*P* = 0.02) were significantly linked with positive test rate. Case fatality rates were associated and a significantly predicted by male/female ratio of the population (*P* = 0.033), median age (*P* = 0.005), health expenditure per capita in USD (*P* = 0.008), NCD mortalities per 100 K (*P* = 0.003), alcohol consumptions per capita in liter (*P* = 0.001), and tobacco use by women (*P* = 0.007). Moreover, case recovered rates were also significantly related to median age (*P* = 0.004), health expenditure per capita in USD (*P* = 0.018), and NCD mortalities per 100 K (*P* = 0.038).

**Table 2:** One to one linear regression analysis using ungrouped independent variables.

	**Positive test rate**				**Case fatality rate**				**Case recovered rate**			
	***R*^**2**^**	***F***	**Regression coefficients (β)**	***p***	***R*^**2**^**	***F***	**Regression coefficients (β)**	***p***	***R*^**2**^**	***F***	**Regression coefficients (β)**	***p***
Male/Female ration	0.001	0.059	−0.027	0.808	0.051	4.694	−0.225	*0.033*	0.035	3.109	−0.186	0.081
Population density/km^2^	0.003	0.29	−0.058	0.592	0.029	2.667	−0.171	0.106	0	0.026	0.017	0.873
Urban population, %	0.001	0.067	−0.028	0.797	0.011	0.994	0.105	0.321	0.035	3.137	0.187	0.08
Median age, years	0.091	8.478	−0.301	*0.005*	0.086	8.393	0.294	*0.005*	0.092	8.787	0.303	*0.004*
PC GNI (PPP) in USD	0.035	3.063	−0.186	0.084	0.003	0.254	0.053	0.616	0.024	2.133	0.155	0.148
PC health expenditure in USD	0.013	1.123	−0.114	0.292	0.076	7.325	0.276	*0.008*	0.062	5.775	0.249	*0.018*
Obesity prevalence, %	0	0.032	0.020	0.859	0.009	0.793	0.096	0.376	0.006	0.501	0.077	0.481
Insufficient physical activity prevalence, %	0	0.008	−0.011	0.929	0.003	0.234	0.056	0.63	0.018	1.341	−0.133	0.251
NCD mortality per 100 K	0	0.005	0.008	0.945	0.099	9.236	−0.315	*0.003*	0.051	4.453	−0.226	*0.038*
Diabetes prevalence, %	0.005	0.425	0.071	0.516	0.008	0.666	−0.087	0.417	0.013	1.162	−0.115	0.284
Alcohol consumptions per capita per L	0.042	3.659	−0.205	0.059	0.11	10.767	0.332	*0.001*	0.04	3.554	0.199	0.063
Tobacco used by male %	0.097	8.95	−0.312	*0.004*	0	0.042	−0.022	0.838	0	0.001	−0.004	0.073
Tobacco used by female %	0.064	5.645	−0.252	*0.02*	0.081	7.678	0.285	*0.007*	0.017	1.522	0.132	0.221

*Linear regression analysis was conducted as one to one analysis with dependent and independent variable. Significant value was calculated as P ≤ 0.05 and mentioned in italic in the table*.

The three dependent variables of the present study, covering 91 countries have mean percentages of positive cases per test, case fatality, and case recovery of 9.94 ± 1.25%, 4.26 ± 0.38%, and 44.98 ± 2.80%, respectively. The goal was to find any significant differences among these variables when countries were grouped according to different socio-economic, demographic, lifestyle, and health determinants.

### Effect of Socioeconomic and Demographic Factors on the Percentage of Positive Tests

**Figure 1:**
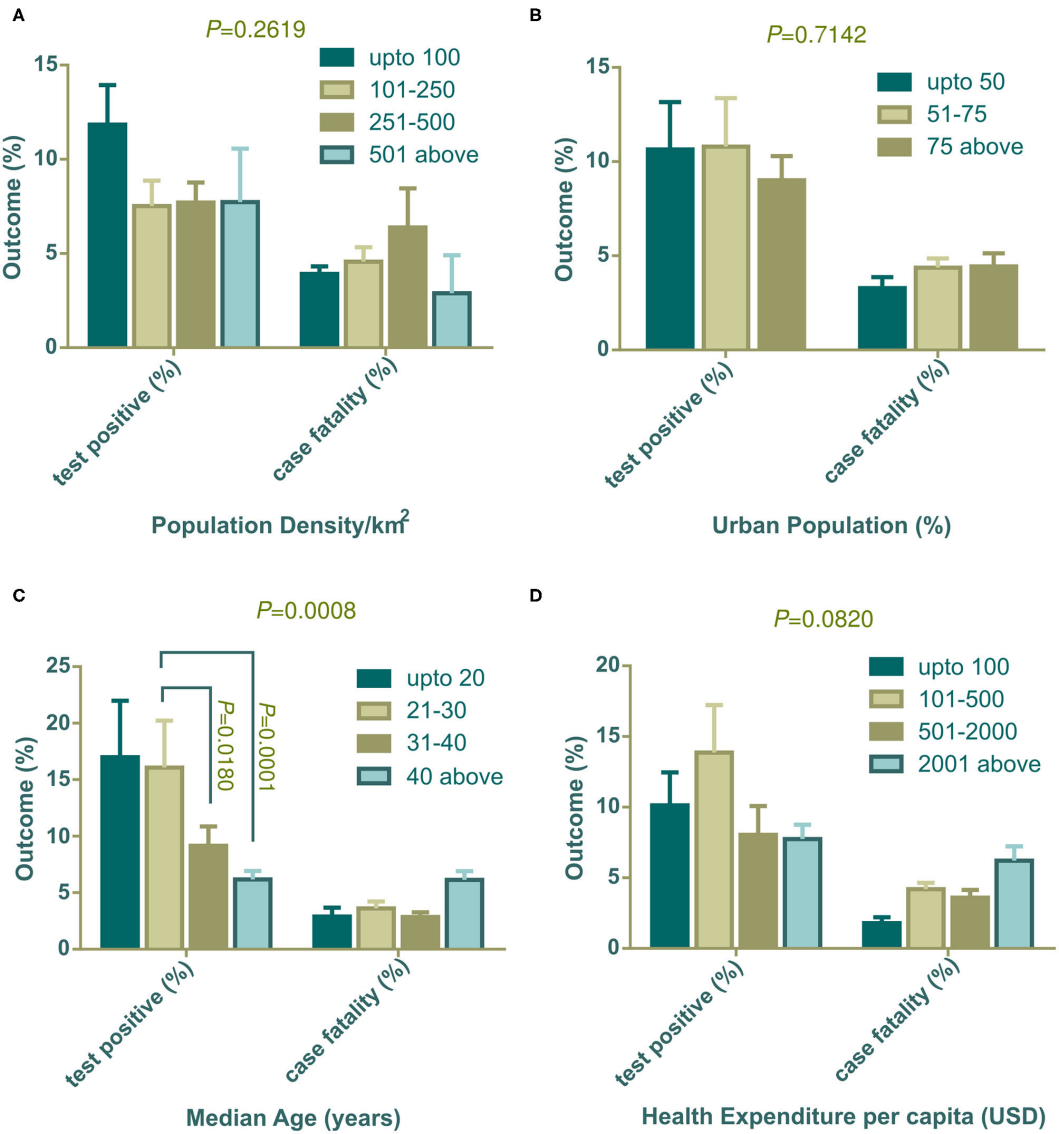
Positive tests (%) and case fatality (%) distribution among countries categorized with population density **(A)**, urban population **(B)**, median age **(C)**, and health expenditure per capita **(D)**. All outcomes (%) are presented as mean ± SEM. In **(C)**, mean positive test (%) of countries with 21–30 years of median age is statistically significant against 31–40 years of median age (*P* = 0.0180) and median age over 40 years (*P* = 0.0001) by Tukey's multiple comparison test. Two-way ANOVA interaction *P-*value are shown on top of each graph. Statistically significant interaction *P-*value indicates that change in independent variable interacts differently on two dependent outcomes.

The population density, percentage of the urban population, median age, and health expenditure per capita were used as independent variables. These variables were grouped as mentioned in Table 1. Data on the total tests performed were not available for four countries (Cameroon, China, Guinea, and Sudan), therefore 87 countries were included in this section. Results showed that countries with low population density (below 100 people per km^2^) had a high percentage of positive tests (Figure 1A, left panel). However, countries in other population density groups had lower positive test rates with minimum variability among them. The percentage of positive tests were slightly lower in countries with high urban density (75% above) compared to other groups (Figure 1B, left panel). Countries with increasing median age showed a statistically significant decrease in the percentage of positive tests (Figure 1C, left panel). The relation between the percentage of positive tests and health expenditure per capita showed no significance regardless of whether country spending had a higher or lower outcome (Figure 1D, left panel).

### Effect of Socioeconomic and Demographic Factors on the Case Fatality Rate

The percentage of case fatalities and its association with population density was uneven and not statistically significant (Figure 1A, right panel), however, countries with a higher urban population showed higher case fatalities (Figure 1B, right panel). Although the percentage of positive tests had a significant association with median age, case fatalities did not show a trend (Figure 1C, right panel). But, countries with the highest number of older populations (median age over 40 years) had a higher fatality rate compared to the other three groups. Similarly, case fatality was found to be high in the countries where the government disburses more in the health sector per capita (Figure 1D, right panel).

### Effect of Socioeconomic and Demographic Factors on Case Recovery Rate

**Figure 2:**
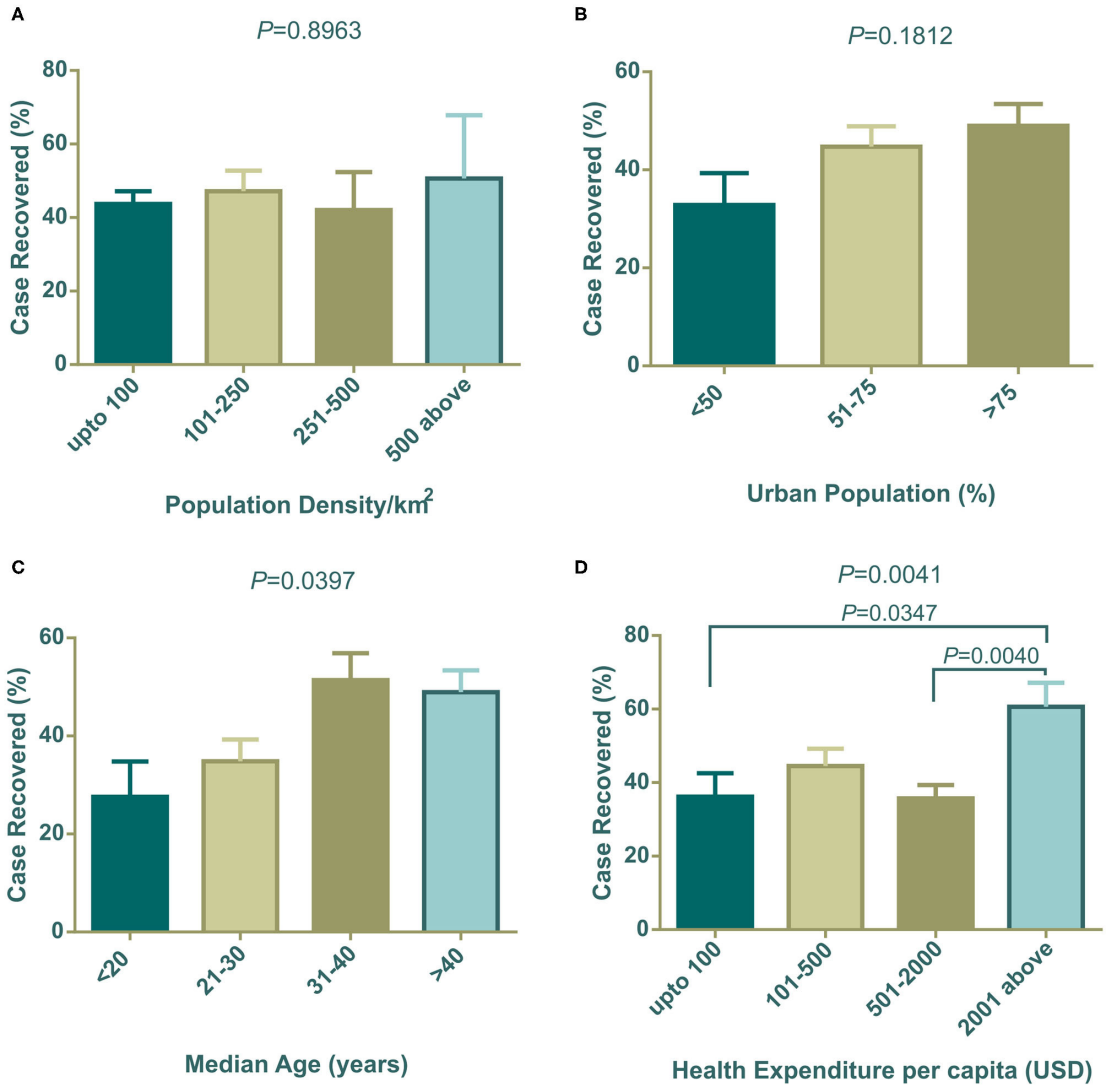
Case recovery (%) distribution among countries categorized with population density **(A)**, urban population **(B)**, median age **(C)**, and health expenditure per capita **(D)**. One way ANOVA statistical differences among column means are shown as *P-*values on top of each graph. In **(C,D)**, differences among means are statistically significant. Data presented are mean ± SEM.

The association of socioeconomic and demographic determinants with the case recovery rate is shown in Figure 2. Population density did not show any connection with the case recovery rate (Figure 2A). Similar to the case fatalities trend, countries with a high urban population showed a higher recovery rate than the countries with a lower dense city population (Figure 2B). Similar to the results for case fatalities, countries with increasing median age showed an increased recovery rate (Figure 2C) and variances of mean values were statistically significant by one-way ANOVA (^*^P = 0.0397). Figure 2D indicates that countries that spend more on healthcare systems per capita, had more recovery rates from COVID-19 (^**^P = 0.0041) (Figure 2D). Due to the unavailability of total recovery case information for the Netherlands and the United Kingdom, they were not included in this segment of analysis.

### Effect of Number of Tests on the Rate of Positive Tests, Fatalities, and Recovery

All countries included in the study varied in COVID-19 diagnosis capacity for social, economic, and political reasons. Taking this into account, we aimed to further examine whether the number of tests performed per 100 K population had any significant effect on mean positive cases, percentage of case positives, case fatality, and case recovery. To do this analysis, we calculated the country specific number of positive cases per 100 K of the population, and tests performed per 100 K population from data on the number of total tests (27), total positive cases (27), and the total population of the country (36).

**Figure 3:**
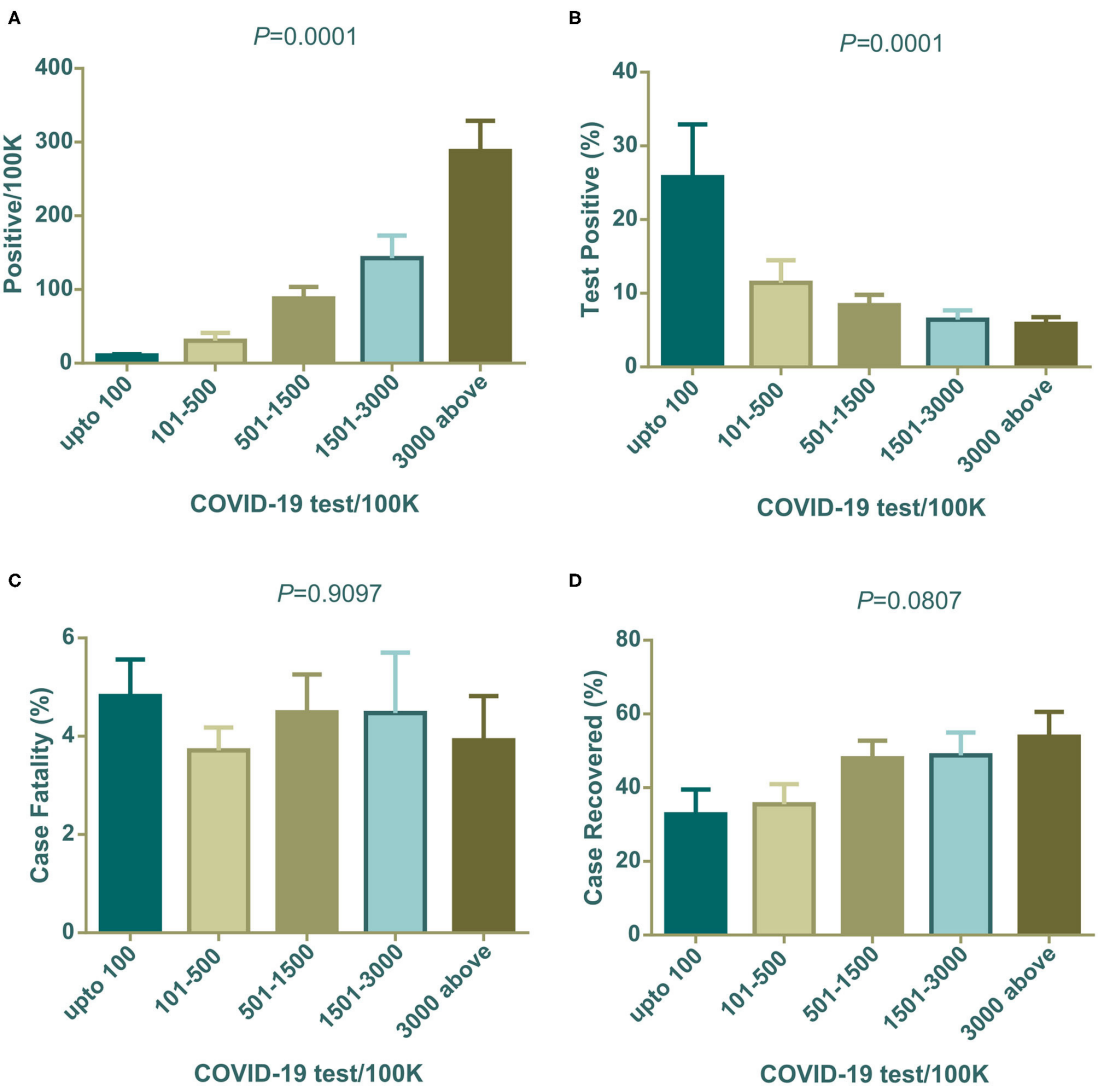
Positives/100 K, positive test (%), case fatality (%), and case recovery (%) data distribution among countries grouped with COVID-19 test/100 K population. All are mean ± SEM. In one-way ANOVA variance among column means are statistically significant for **(A,B)**. Case fatality (%) and case recovered (%) were not statistically significant **(C,D)**. One way ANOVA differences among column means are shown as P-values on top of each graph.

The association of COVID-19 test numbers per 100 K population is presented in Figure 3. Positive cases per 100 K of the population were significantly (^****^P < 0.001) boosted with an increased number of tests performed per 100 K population (Figure 3A), in contrast, the percentage of positive tests were high in the lowest COVID-19 tests per 100 K populations (^****^P < 0.0001) (Figure 3B). Case fatality did not change significantly among countries grouped into increasing test numbers (Figure 3C). Higher case recovery rates were observed in the groups of countries where a higher number of tests were performed (Figure 3D). Cameroon, China, Guinea, and Sudan were out of this analysis due to the unavailability of test data from these countries.

### Lifestyle and Health Indicators

Individual, community, and/or social lifestyle can influence the transmission of viruses as well as death and recovery rates. Another study has shown that health indicators like the prevalence of diabetes and other NCDs also influence the number of death and recovery (46). Therefore, we considered the prevalence of obesity, insufficient physical activities, diabetes, NCDs mortality per 100 K population, consumption of alcohol per capita per liter per year as well as the percentage of general tobacco uses in male populations as independent variables.

#### Lifestyle and Health Indicators on Case Fatalities of COVID-19

**Figure 4:**
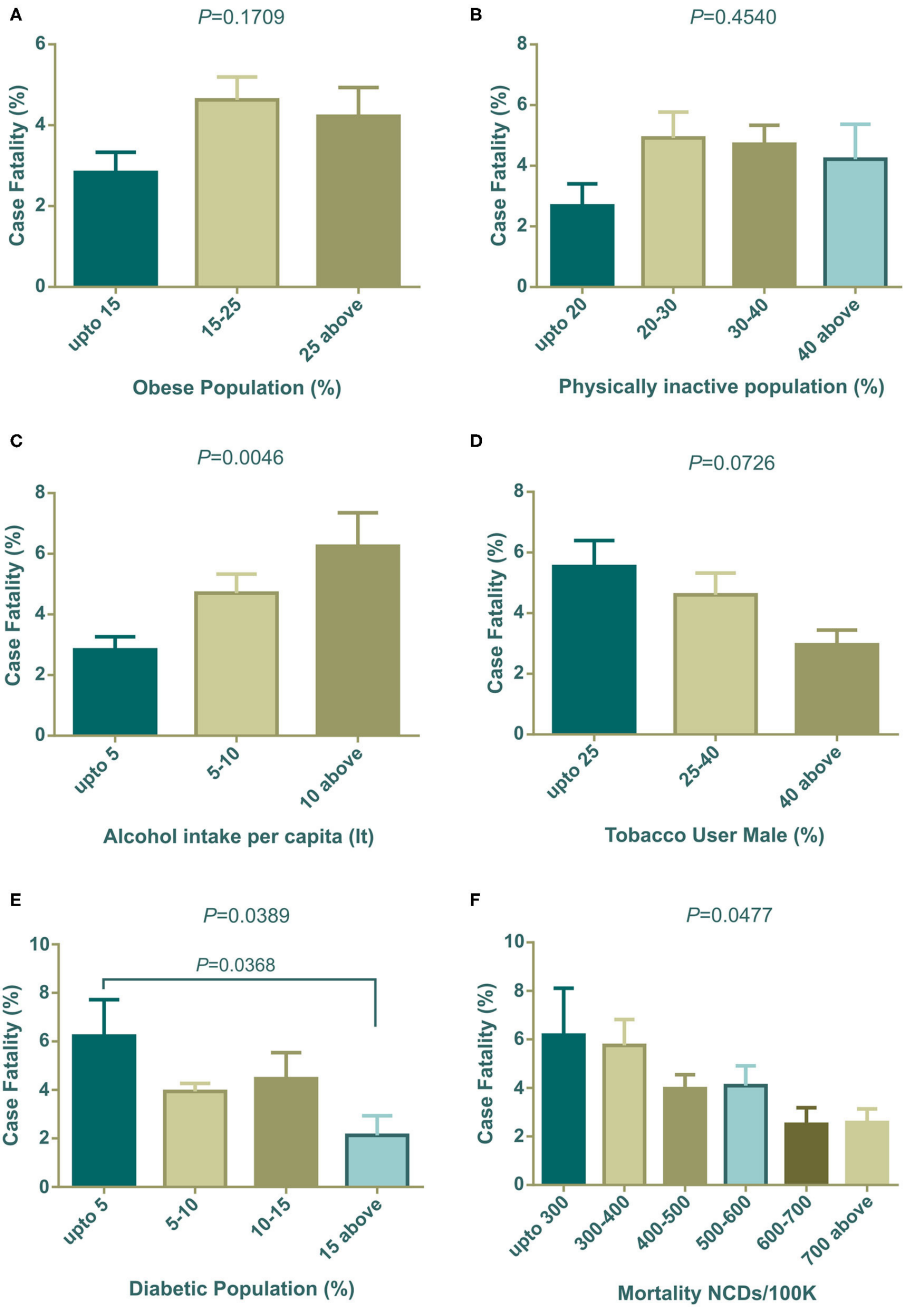
COVID-19 case fatality (%) distribution based on countries grouped according to health indicators **(A–F)**. Alcohol intake, diabetic population, and NCDs mortality/100 K were statistically significant by one way ANOVA. Data presented are mean ± SEM.

In our investigation, countries with high obesity prevalence were witnessing more COVID-19 related deaths (Figure 4A) and correspondingly, countries where people were less physically active also had increased death rates (Figure 4B). Countries with high alcohol consumption were significantly (*P* = 0.0046) prone to death (Figure 4C). However, interestingly, tobacco use patterns showed different scenarios compared to alcohol consumption. Higher percentages of any kind of tobacco used in males showed fewer case fatalities compared to other groups of countries (Figure 4D). Surprisingly, countries with low diabetes prevalence showed a significantly (*P* = 0.0389) higher death rate (Figure 4E). NCD mortality per 100 K population showed a negative relation with the COVID-19 case fatality rate (*P* = 0.0477), and countries with high NCD mortality experienced less COVID-19 case fatality (Figure 4F).

#### Lifestyle and Health Indicators on Case Recovered Rate of COVID-19

**Figure 5:**
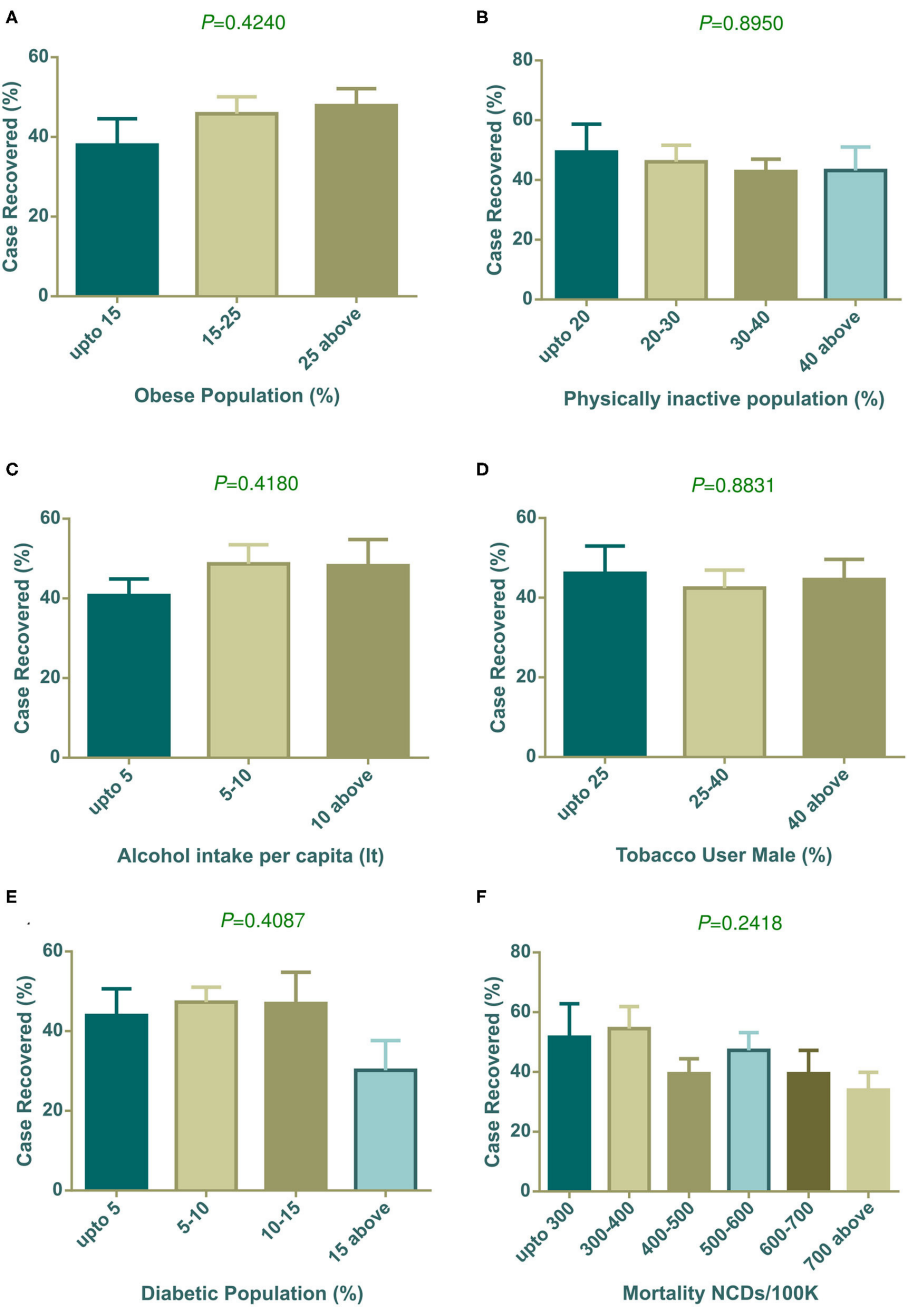
COVID-19 case recovery (%) distribution based on countries grouped according to health indicators **(A–F)**. None of the parameters showed statistically significant variation among column means by one way ANOVA. Data presented are mean ± SEM.

Our analysis (Figure 5A) showed that countries with an increasing prevalence of obesity were related to the COVID-19 case recovery rate. Insufficient physical activity did not show any meaningful association with the recovery rate (Figure 5B). However, high alcohol consumption displayed a relationship with high recovery, though it was not statistically significant (Figure 5C). The tobacco use pattern did not show any trends (Figure 5D). The highest prevalence of diabetes exhibited a low case recovery rate (Figure 5E) compare to other groups of countries. Low NCD mortalities per 100 K groups had a relatively high case recovery rate (Figure 5F).

### Parametric Distribution of Countries With Top Case Fatality Rates

**Figure 6:**
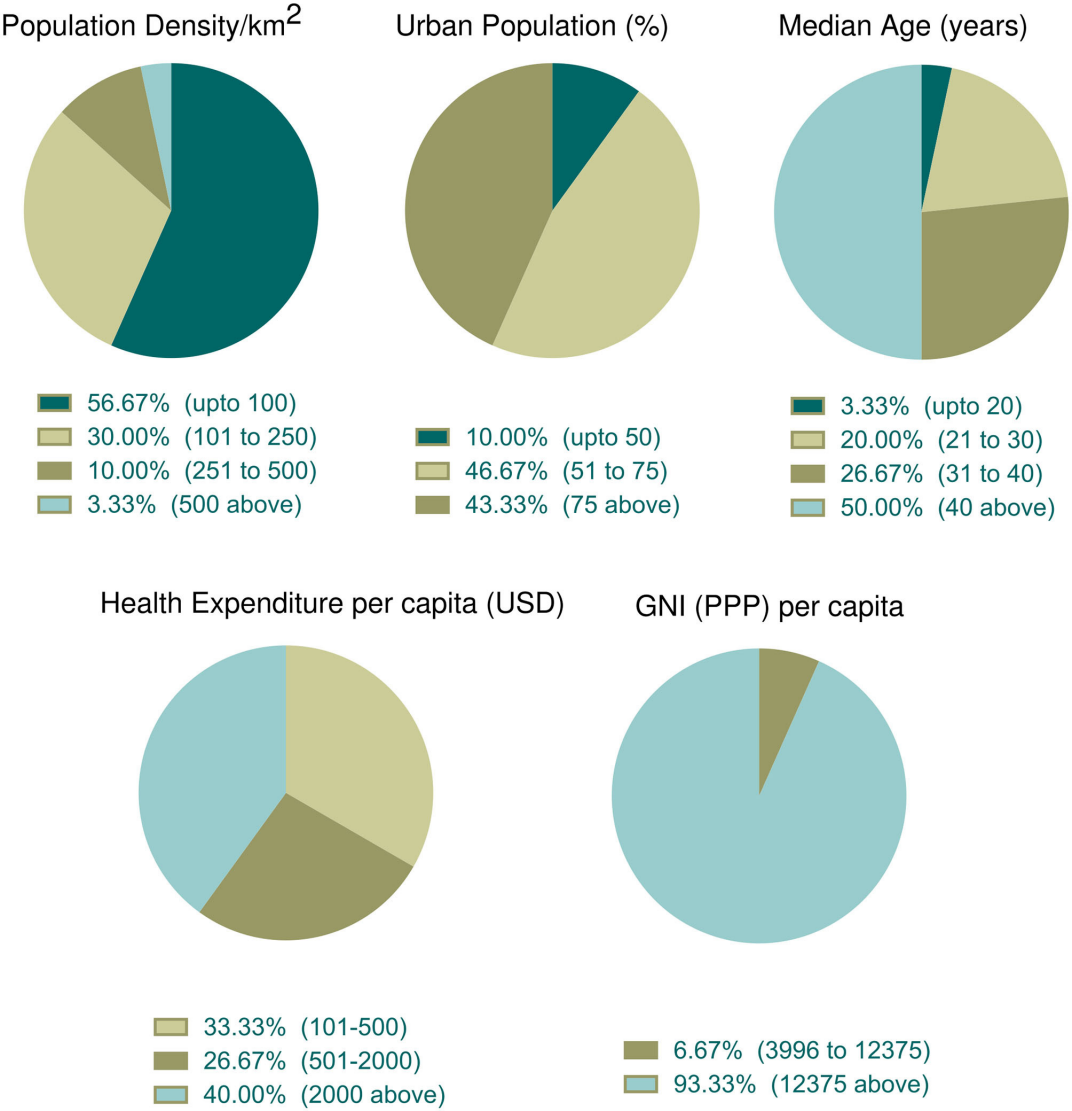
Incidence of socioeconomic and demographic parameter distribution among the top thirty countries with high case fatality (%). Data categories with zero data are not shown in the pie chart.

**Figure 7:**
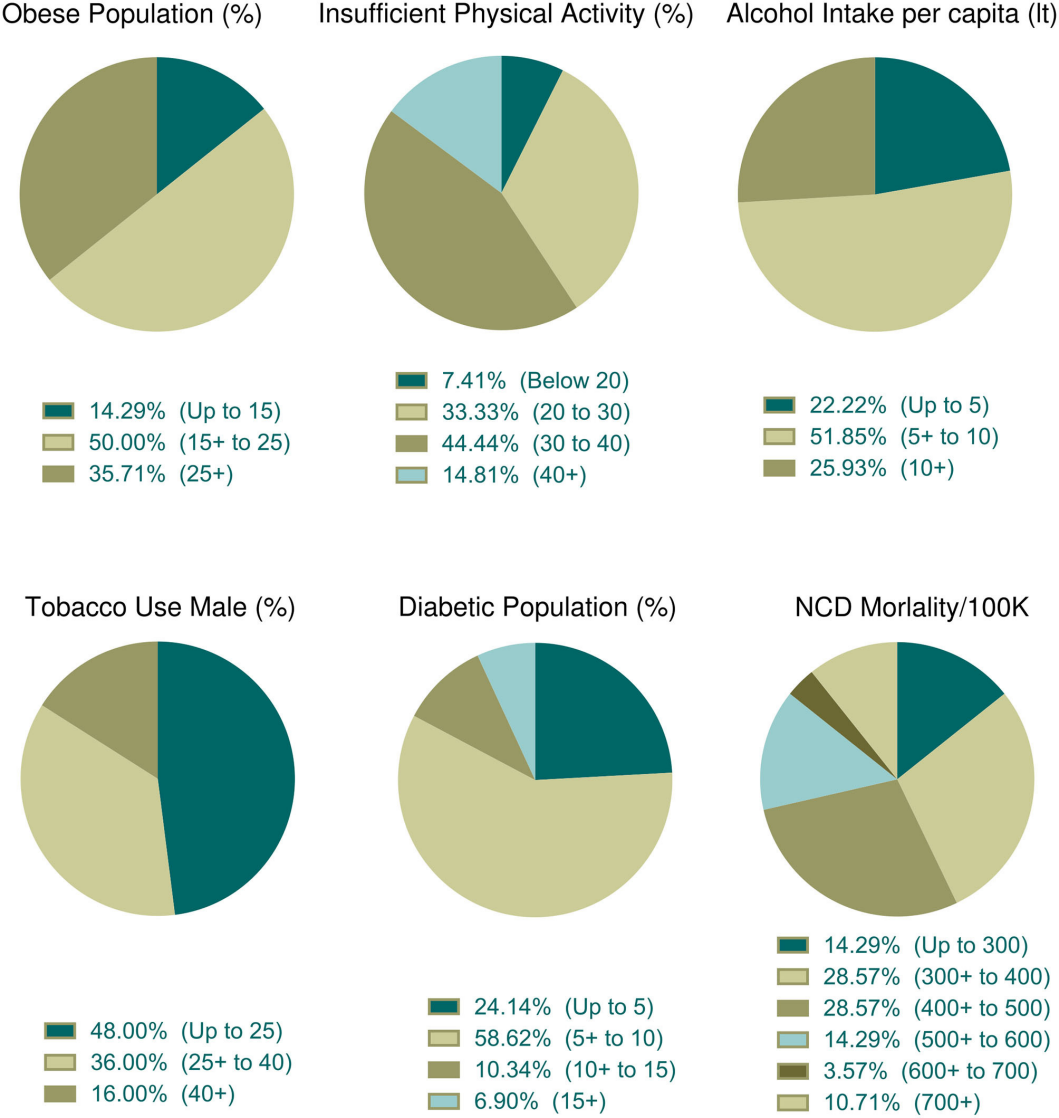
Distribution of health indicators incidence among the top thirty countries with high case fatality (%). Data categories with zero data are not shown in the pie chart.

The association of different factors with dependent variables showed a mixed pattern. In this context, we wanted to further explore countries with high fatality rates so that parametric trends can be observed. To do that, we selected 30 countries with more than 5% case fatalities from among 91 countries (Table 3). The mean ± SEM values of all parametric distributions are also shown in Table 3. Individual parameter distributions are shown as pie charts in Figures 6, 7.

**Table 3:** Top 30 countries according to case fatality rate with their parametric distributions.

**Country**	**Per capita GNI (PPP), USD**	**Population density, KM^**2**^**	**Urban population Percentages**	**Median age, years**	**Per capita health expenditure, USD**	**Prevalence of obesity, %**	**Prevalence of insufficient physical activity, %**	**NCD mortality per 100 K people**	**Prevalence of diabetes, %**	**Alcohol consumption, per capita in litter per year**	**Prevalence of any kind of tobacco use by male, %**
Sweden	54,030	25.00	87.43	41.10	5904.58	8.6	NA	745	22.1	0	0
Slovenia	37,450	102.64	54.54	44.90	1920.28	27.4	33.6	446.6	6.7	0.6	0
Mexico	19,340	64.91	80.16	29.30	494.68	22.1	47	452	14.2	6.5	0
Greece	29,670	83.22	79.06	45.30	1516.59	18.9	11.5	768	5.7	9.3	0
Denmark	56,410	138.07	87.87	42.00	5800.15	19.9	27.2	405.1	5.5	3.3	12.3
USA	63,690	35.77	82.26	38.50	10246.14	19.7	28.5	356.6	8.3	9.5	13.4
Brazil	15,850	25.06	86.57	33.20	928.80	29.4	28.6	291.6	7.6	8.1	14.8
Algeria	14,970	17.73	72.63	28.90	258.49	28.9	28.9	457.8	13.5	5.5	17.3
Philippines	10,740	357.69	46.91	24.10	132.90	27.8	35.9	342.4	3.9	9.8	17.3
Canada	47,590	4.08	81.41	41.80	4754.95	36.2	40	417.9	10.8	8.8	17.4
North Macedonia	15,670	82.59	57.96	39.00	328.42	NA	23.1	318.3	4.8	7.2	17.5
Hungary	29,860	107.91	71.35	43.60	981.42	25.8	33.2	560.8	9.6	0	19.4
France	46,360	122.34	80.44	41.70	4379.73	25.3	32.7	348.2	3.2	11.3	20
Ecuador	11,420	68.79	63.82	28.80	518.03	20.2	32.2	352.4	5.9	10.8	20.4
Indonesia	12,670	147.75	55.33	31.10	114.97	22.1	35.7	333.5	4.6	10.4	24.2
Ireland	67,050	70.45	63.17	37.80	4976.86	19.5	23.7	285.4	5.7	9.5	24.7
Belgium	51,740	377.21	98.00	41.60	4507.36	28.3	41.6	424.8	5.9	8.4	26
Sudan	4,430	21.30	34.64	18.30	193.79	19.9	41.4	306.4	5	7.1	26.9
Honduras	4,790	85.69	57.10	24.40	195.94	21.6	29.3	290	4.8	11.8	27.7
United Kingdom	45,350	274.83	83.40	40.60	3858.67	21.4	NA	442.4	7.3	2.9	27.8
China	18,170	148.35	59.15	38.40	440.83	23.8	26.8	297.4	6.9	8.5	28
Argentina	19,870	16.26	91.87	32.40	1324.60	26.4	38.5	580.2	6.9	10.9	28.3
Switzerland	68,820	215.52	73.80	42.70	9956.26	23.1	32.5	467.8	6.1	10.5	28.9
Netherlands	56,890	511.46	91.49	42.80	4911.44	22.5	35.4	577.2	6.9	10.4	33.1
Poland	30,010	124.04	60.06	41.90	906.82	6.4	39.7	678.3	7.1	4.5	39
Spain	39,800	93.53	80.32	43.90	2506.46	6.2	14.1	542.4	9.2	5.7	45.7
Italy	42,290	205.45	70.44	46.50	2840.13	24.9	37.7	340	4.7	6.4	49.7
Iran	21,050	50.22	74.90	31.70	475.48	32	31	826.7	17.2	0.2	56.3
Egypt	12,100	98.87	42.70	24.10	105.77	6.9	22.6	NA	6.3	0.3	82.7
Romania	27,520	84.64	54.00	42.50	555.10	NA	NA	NA	NA	NA	NA
Mean ± SEM	32520.00 ± 3573.60	125.38 ± 21.62	70.76 ± 2.91	36.76 ± 1.40	2534.52 ± 516.47	21.97 ± 1.41	31.57 ± 1.56	451.97 ± 29.21	7.81 ± 0.78	6.83 ± 0.71	24.79 ± 3.31

*Data sources are mentioned in methodology and in supplements. Countries were selected decisively whose case fatality rates were above 5%. NA = Data not available*.

#### Socioeconomic and Demographic Distribution Among Countries With Top Case Fatality Rates

Socioeconomic and demographic distribution among the top 30 countries whose case fatality rates are more than 5% were illustrated in Figure 6. According to data, more than half of the top 30 countries (*n* = 17, 56.67%) had the lowest population density per km^2^. The percentage of the urban population was high (above 50%) in 90% of countries (*n* = 27). Half of these countries had an older population (median age over 40 years). About 40% of countries (*n* = 12) spent more money in health sectors per capita and 93.33% (*n* = 28) of countries belonged to the high income group.

#### Lifestyle and Health Parameter Distribution Among Countries With Top Case Fatality Rates

The prevalence of obesity was observed in 28 countries. Among them, 50% of countries belong to a moderate percentage of the obese population (Figure 7). Physical activity data were available for 27 countries and among them, 44.44% (*n* = 12) of countries indicated that 30–40% of people were physically inactive (Figure 7). About 51.85% (*n* = 14) of countries among the 27 consumed 5–10 liters of alcohol per capita per year and nearly half of these countries (*n* = 12 of 25, 48%) used fewer tobacco products (Figure 7). About 58.62% of countries (*n* = 27 of 29) were moderately diabetic (5–10% diabetic prevalence) and more than half (*n* = 16 of 28, 57.14%) of the countries were at risk of death, whose NCDs mortality rate per 100 K population was low to moderate (Figure 7).

## Discussion

### Socioeconomic and Demographic Determinants of COVID-19 Positive Tests, Case Fatality, and Case Recovery Rate

Population density both in a country and in urban cities can have an impact on contagious disease transmission. Both contact rates and patterns in a defined geographical spatial distribution determine the mode of infectious disease transmission (47, 48). The contact hours and contact number per person varies with age and number of household members. In a study in Hong Kong, the highest contact pattern was observed among school-going children, which decreased with age (48). However, the older age group with economic strength also showed elevated contact rates. We found a weak relation of population density per km^2^ with the percentage of positive tests (Figure 1A). Generally, population density poses an increased chance of disease transmission, but the contact rate is not always determined by population density. At a low density, contact rate can increase rapidly which gets saturated in a very dense area where lack of organized social contact is evident (47). At this point, the contact rate becomes independent of population density. Although in our analysis low density countries show a slightly higher percentage of positive tests, further data analysis showed that few countries in this group e.g., Algeria, Brazil, and Afghanistan had very high positive test value as outliers as they did a very low number of tests. This inadequate testing increased their positive test rate, which agrees with our analysis in this study (Figure 3B).

The number of tests performed to confirm COVID-19 is a challenging issue for many countries and can affect decision making drastically. We found a statistically linear positive relation in the number of positive patients, with increasing test numbers (Figure 3A). The following graph depicted the number of tests per 100 K population, showing that as they increased the percentage of positive tests dropped gradually (Figure 3B). In a recent press conference, the WHO discussed that a high percentage of positive tests in a country indicates that an inadequate number of tests were being performed, and they set a rough standard of 10% positive tests as an indicator of normal outcome (49). This strongly supports our data in Figure 3B. Countries that performed above 500 tests per 100 K population, showed around 10% positive tests. Countries with lower middle and upper middle economies performed symptom-based highly selective diagnoses due to socioeconomic, demographic, and political reasons which increased the percentage of positive tests.

Positive test rates also have close links with the urban population, median age, and income level. Among 91 countries in our study, around 74.73% (*n* = 68) of countries belonged to the high economy class, 20.88% (*n* = 19) and were from the upper middle economy and the rest were from the lower middle economy group (Table 1). Thus, most of the independent socioeconomic and demographic variables comply with the characteristics of developed countries. These countries possess modern health facilities, medical professionals, and cutting-edge research facilities. Despite having these benefits in health sectors they could not successfully restrict infections and deaths toll. However, these amenities certainly were advantageous in some form and contributed to patient recovery (Figure 2D).

Developed countries have larger city populations with a greater proportion of older inhabitants who prefer to stay at home (50, 51). The positive test rates thus were lower in groups of countries with dense urban populations (Figure 1B). Median age data indicated that developing countries have younger populations compared to developed countries. The young population has tendencies to go outside, making them a vulnerable age group (32). Therefore, positive test rates were high in developing countries where the median age is below 30 years (Figure 1C). Health expenditure per capita correlates with a higher number of tests being performed, thus countries with over 501 USD investments showed a decreased positive test rate (Figure 1D). As high median age belongs to countries with a strong healthcare system, case recovery was also found to be high where the median age is over 30 years (Figure 2C).

Case fatality and recovery from epidemics largely depend on internal and external factors along with age, presence of co-morbidities, health facilities, and the pattern of adherence at healthcare centers (25, 33, 50, 52). The effect of contagions on certain populations is also influenced by the interplay between the incubation period and the age dependent case fatality rate of the disease (53). In our study, the case fatality rate was high where the median age is over 40 years (Figure 1C) and in countries with more than 50% urban population (Figure 1B). Although increasing population density showed a gradual rise in case fatality, countries with over 500 people per km^2^ density (*n* = 6), surprisingly, showed a reduced case fatality (Figure 1A). Most of the countries in this group have a high urban population and high median age. However, they managed to control case-fatality with remarkable success, except in the Netherlands, where they had a 12.79% case fatality. With rapid urbanization, the risks of pandemics and zoonotic diseases are increasing (54). Because it is an infectious and highly contagious disease, urban population percentages are one of the most important predictors in COVID-19 outcome. Countries with more people living in rural areas have lower case fatalities as air velocity potentially reduces disease transmission (55). In contrast, in urban medical facilities, the availability of medicines/drugs, and treatment tools accelerate disease recovery, despite a large number of patients.

Countries with high health expenditure per capita and GNI (PPP) could not restrict the pace of death as expected due to inadequate intensive care equipment and management personnel, and an overwhelming number of critical patients. Although better urban intelligence and management are partially linked with better recovery (56), as the proportion of the urban population around the globe is gradually increasing, a policy level rethink of the healthcare system is vital. We need to redesign urban intelligence, resource management, and coordination to fight the COVID-19 pandemic, in circumstances where isolation, social distancing, and quarantine are challenging (57).

Investment in health, especially toward NDCs, have shown reduced mortality/incidence ratio in cancer (58), stroke (59), and child mortality (60). Expenditure to contain the infectious disease is still a necessary component; as the controlled use of available resources can reduce the disease spreading (61). Moreover, sudden pandemic induced resource constraints can critically affect treatment and patient recovery (62). Contrary to misconceptions that higher expenditure relates to better healthcare, developed countries showed no strong negative correlation between health expenditure and case fatality (63). This could be due to the inappropriate ways in which they spend money, poorly designed policy, and political intrusions that inhibit policy and treaty implementation (64). During the COVID-19 pandemic, some countries have offered strict preventive measures and information on technology-based contact tracing to successfully contain the disease, although they fall into high risk group countries. In our study, COVID-19 related case fatality rate was comparatively higher in countries with high health expenditure per capita, which explains that the majority of health investment is insufficient or inadequate in required segments to tackle such a pandemic. In a recent study, COVID-19 incidence and case fatality was found not to be associated with health expenditure and services (34). The situation gets worse as the incidence number rises rapidly, putting pressure on healthcare capacity, as observed in some European countries (25).

### Lifestyle and Health Indicators on the Case Fatality Rate

The duration of infectious disease is very important and has a direct impact on mortality (53). Clinical recovery from COVID-19 cases requires ~14–42 days depending upon the patient's physical and clinical conditions (65). Thus, unhealthy lifestyles are important predictors of delayed recovery or death. Lifestyle includes obesity, physical activities, the pattern of drinking alcohol, tobacco usages, etc. and the prevalence of NCDs and their mortality is also linked to the raised number of case fatality rates.

Several factors including obesity, diabetes, cardiovascular disease and hypertension, cancer, and chronic respiratory diseases, have been identified as collective underlying conditions of critical illness that can lead to poor outcomes in COVID-19 (66, 67). Recent studies have indicated that obesity and insufficient physical activity might accelerate the mortality of COVID-19 (66, 67). Our study reflects these results, as it indicated that countries with a large number of obese and insufficiently active populations had an increased number of case fatality rates. However, the Center for Disease Prevention and Control (CDC, USA), listed severe obesity (BMI over 40) as a risk factor for critical COVID-19 cases (68). Furthermore, clinical reports of critical COVID-19 patients have shown that significant numbers of patients are associated with obesity in different countries (66). Obesity and insufficient physical activity can lead to metabolic, cardiovascular disorders, and other NCDs (69). Though the relationships between obesity and NCDs are well-studied, little is known about the effect of obesity on immunity and contagious disease. Recent clinical studies on survivors and non-survivors of COVID-19 have shown that inappropriate and abnormal immunity were significantly associated with death (70). Several animal model studies showed that obesity leads to impairment of natural killer cells, reduction of macrophages, and dendritic cell activities (69), with reduced cytokine productions and weakened responses to antigen stimulations. Thus, impaired immune systems cannot fight the pathogen resulting in delayed recovery or death.

Other studies have showed that diabetes is the second top co-morbidity factor in COVID-19 case fatality after hypertension (66). Surprisingly, our analysis demonstrated that the group of countries with the lowest prevalence of diabetes observed more case fatality (Figure 4E). To explain this outcome, we further analyzed the group prevalence of diabetes with group median age, as diabetes is more prone to elderly people (Supplementary 1, Table 1). The analysis demonstrated that 55.56% (*n* = 10 of 18) countries holding up to 5% diabetes prevalence comprises the median age over 40 years, and none of the countries with 15% diabetes prevalence were from the same median age group of countries. Furthermore, cross analysis with GNI (PPP) per capita (Supplementary 1, Table 2) confirmed that 72.22% of developed countries were from the group with less prevalence of diabetes. This result established that diabetes is one of the key factors in case fatalities, which reflects this the findings of this study, demonstrating that case fatalities were more evident in elderly people and developed countries.

Diabetes is also characterized by chronically elevated levels of blood glucose. These raised blood sugar concentrations also increase glucose concentration in airway secretions (71). Another study also demonstrated that *in vivo* influenza virus infection and replications can be significantly increased due to the contact of pulmonary epithelial cells with raised glucose concentrations *in vitro* (72). Raised sugar levels in the blood could also weaken the anti-viral immune response and can be reverted with insulin treatment (73). Another study showed that high glucose concentration or diabetic conditions were associated with fatal outcomes in avian influenza (74). Therefore, it could be concluded that chronic diabetic conditions can elevate the case fatality rates of COVID-19.

Countries with less NCDs mortality per 100 K population faced more COVID-19 death rates (Figure 4F). To explain this, we again did a cross analysis of group NCDs mortality per 100 K with group median age and group GNI (PPP) per capita. Around 71.43% of the countries of the lowest NCD mortality per 100 K belong to the highest group of median age and all of them were high income group countries (see Supplementary Table 2). As discussed above, case fatalities were high in developed countries and the cross analysis of NCDs mortality with median age and per capita income also suggested that elderly people with NCDs were more at risk of fatal outcomes. Most incidences of mortality (60–90%) were related to preexisting one or more NCDs (75–78).

Heavy alcohol intake has been casually associated with several diseases including infectious disease (79). This could be explained by the fact that heavy intake of alcohol weakens the immune function as well as several organs including the liver and lungs, making them susceptible to microorganisms (79), and substantially lowers the adherence of antiretroviral therapy, accelerating mortality (80). Our investigations showed that case fatality was increased with per capita alcohol intake in liters (Figure 4C).

Tobacco smoking is generally linked with lung diseases (81) and can facilitate microbial infection (82). There is currently an ongoing scientific debate about the relationship between tobacco smoking and severity of COVID-19 (83). A recent systematic review concluded that smoking might be negatively linked to COVID-19 case fatality (84). However, another short meta-analysis stated no association between them (85). In addition, a group of French scientists have shown that tobacco smoking has negative effects of COVID-19 mortality (86) which was also reflected in our results (Figure 4D). Recent news (87, 88) reported that French scientists are planning a human trial to test whether tobacco can fight COVID-19. Due to the fact that this debate is ongoing, a more detailed clinical and molecular investigation is required to establish a more conclusive answer.

### Limitations and Future Directions

The COVID-19 pandemic is ongoing and not yet closed, and the data used in our study reflects a snapshot of a point in time. This limits our study, which cannot capture the full view of the dynamic nature of this disease. Due to limited number of tests, many asymptomatic carriers can be left outside the diagnosis process and positive tests, case fatality and case recovery cannot be estimated accurately. However, we had to exclusively depend on the number of confirmed cases for any calculation. In addition, there are potential reliability issues in terms of the accuracy of this self-reported government data. As COVID-19 is multifactor mediated, not all factors could be included in this particular study, and integrating the molecular mechanism of the disease was beyond its scope. In the future, the nature of this molecular mechanism and its pathogenesis will gradually unfold, and more clinical data will be available, and the factors discussed in this study will be easier to interpret. Finally, the interpretation of this study could be useful in designing future studies and attempts to effectively contain such a contagious pandemic outbreak within a very short time.

## Data Availability Statement

All datasets presented in this study are included in the article/Supplementary Material.

## Author Contributions

AA and MMR conceived and designed the study, conducted the analysis with input from TH, equally contributed to the first draft. MMR and TH collected and sorted the data from different sources. TH added additional points and contributed to its development. After necessary corrections and suggestions from all authors, AA finalized and submitted the manuscript. All authors contributed to the article and approved the submitted version.

## Conflict of Interest

The authors declare that the research was conducted in the absence of any commercial or financial relationships that could be construed as a potential conflict of interest.
