# Supplementary figures and images for: Lifestyle Acquired Immunity, Decentralized Intelligent Infrastructures, and Revised Healthcare Expenditures May Limit Pandemic Catastrophe: A Lesson From COVID-19
Source: Front Public Health. 2020 Nov 5;8:566114. doi: 10.3389/fpubh.2020.566114 (PMC7674625; doi:10.3389/fpubh.2020.566114)

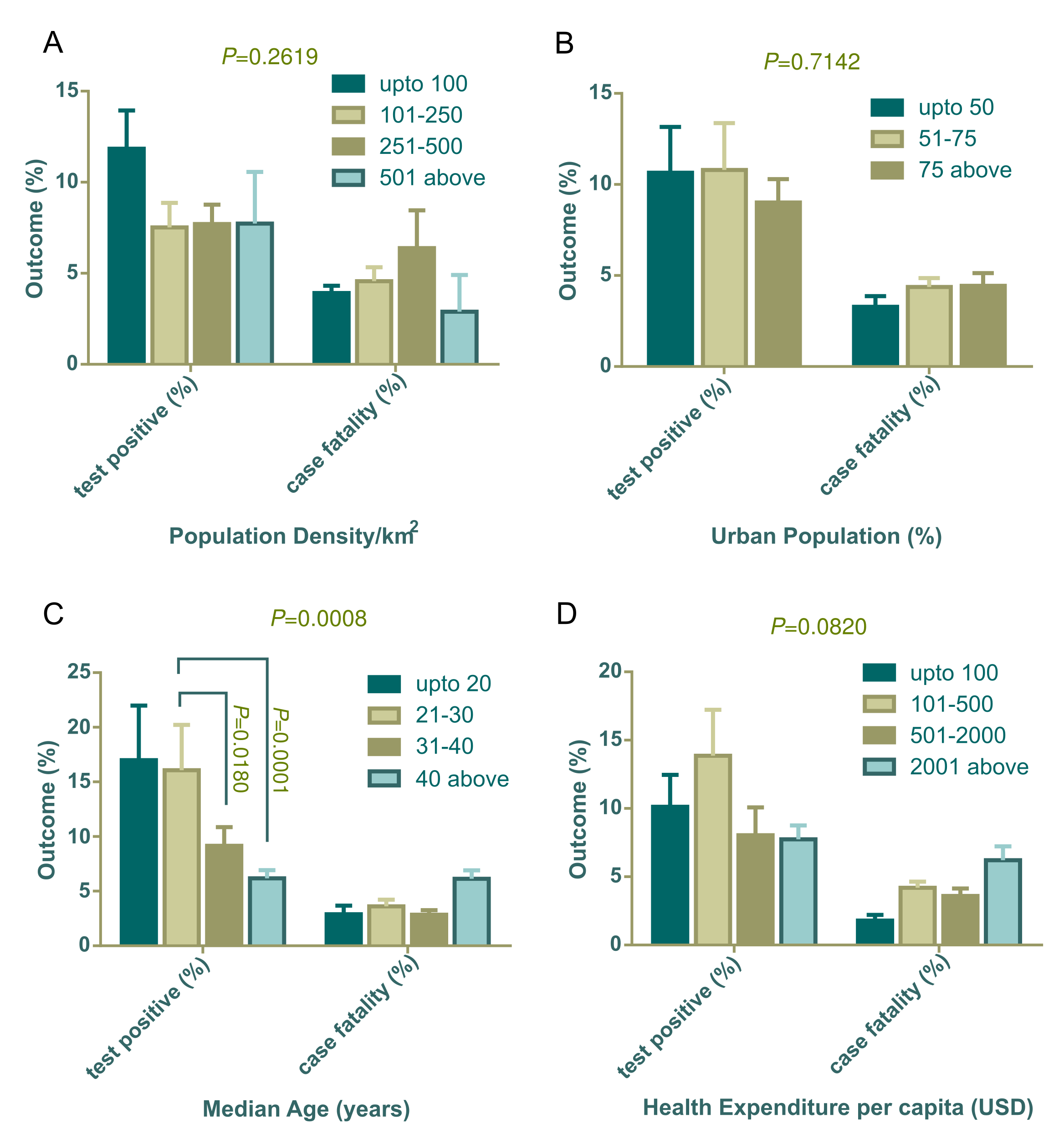

Supplement: Supplementary file 1 [file Figure_1.TIF]

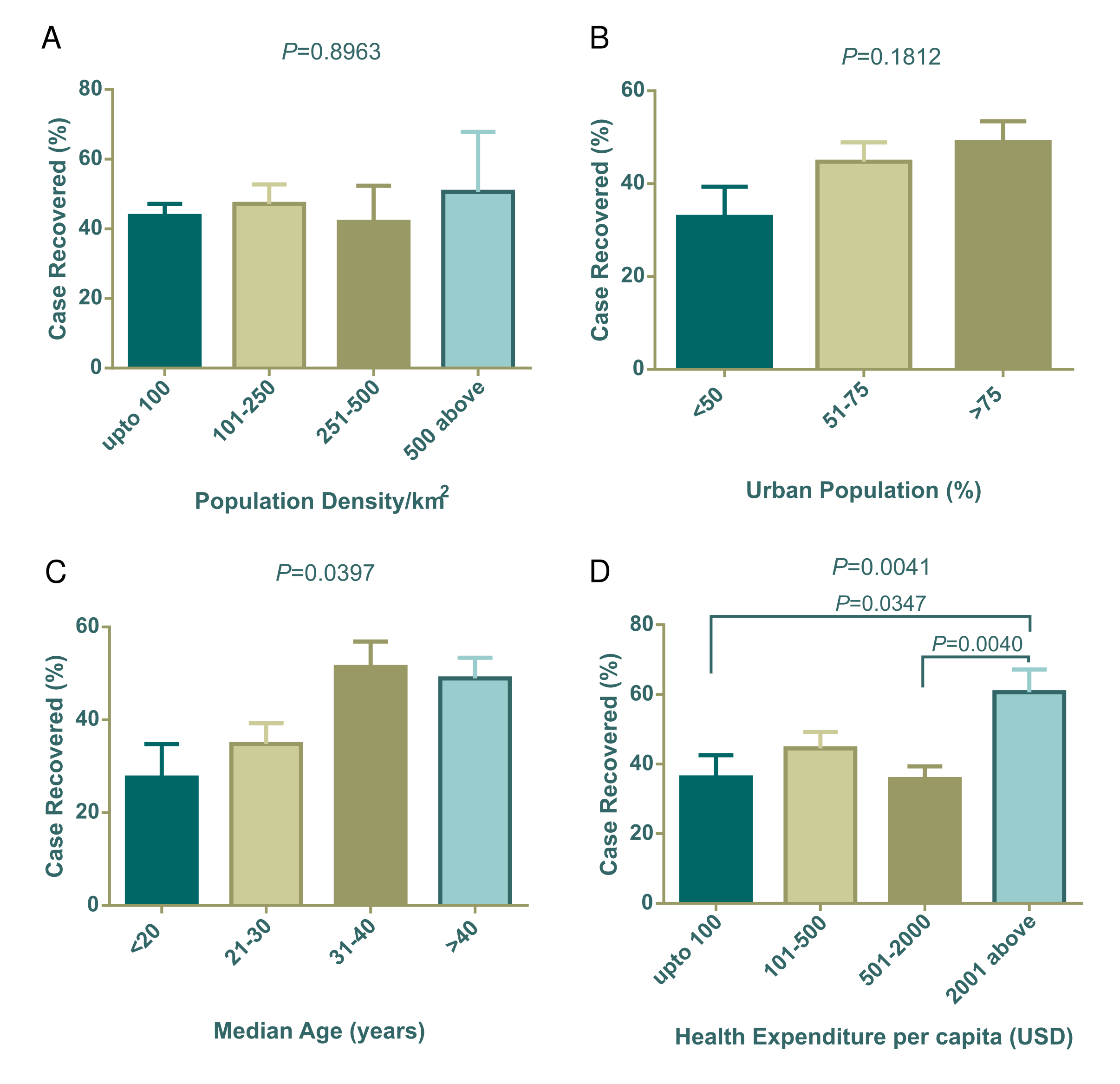

Supplement: Supplementary file 2 [file Figure_2.TIF]

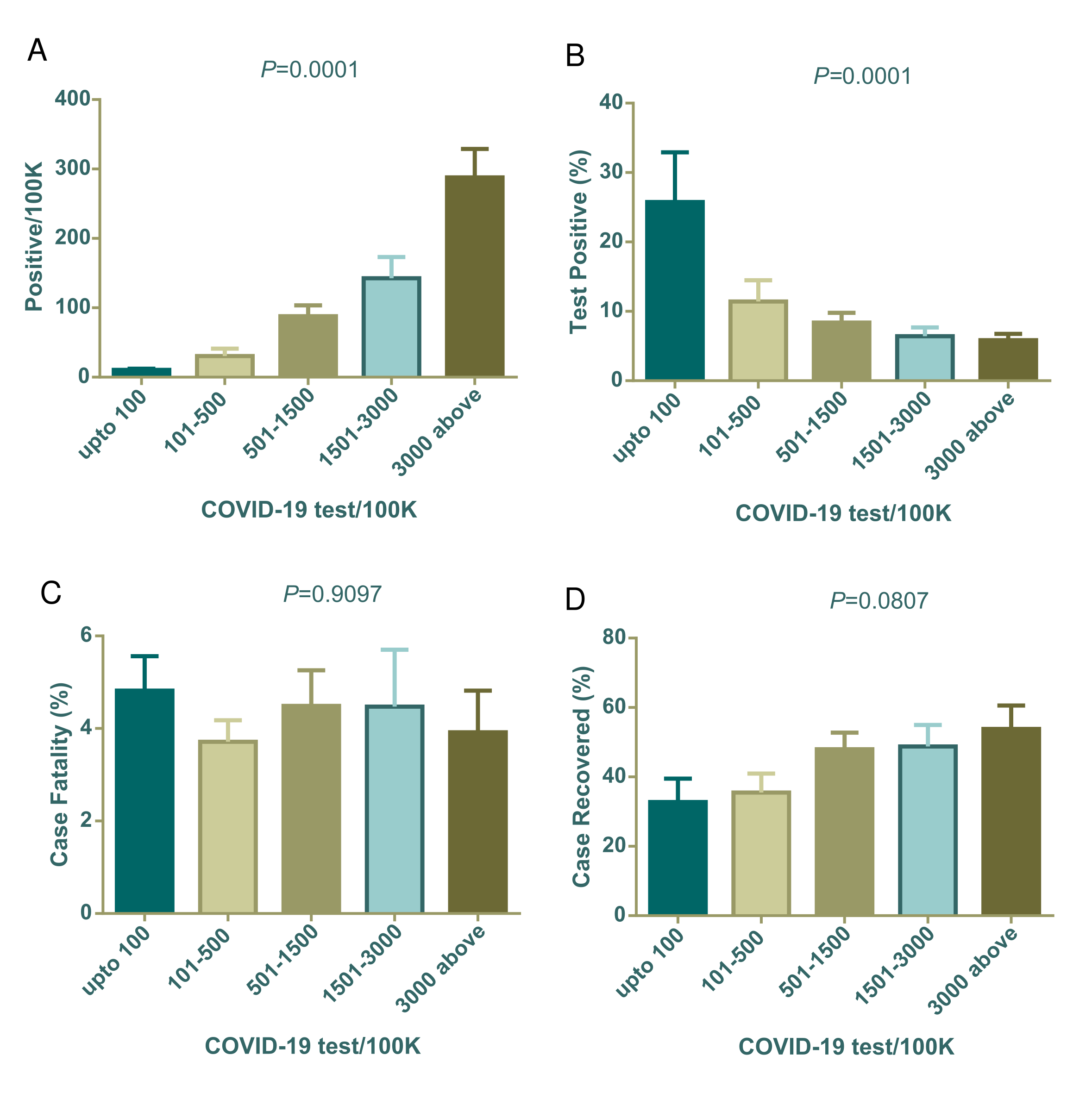

Supplement: Supplementary file 3 [file Figure_3.TIF]

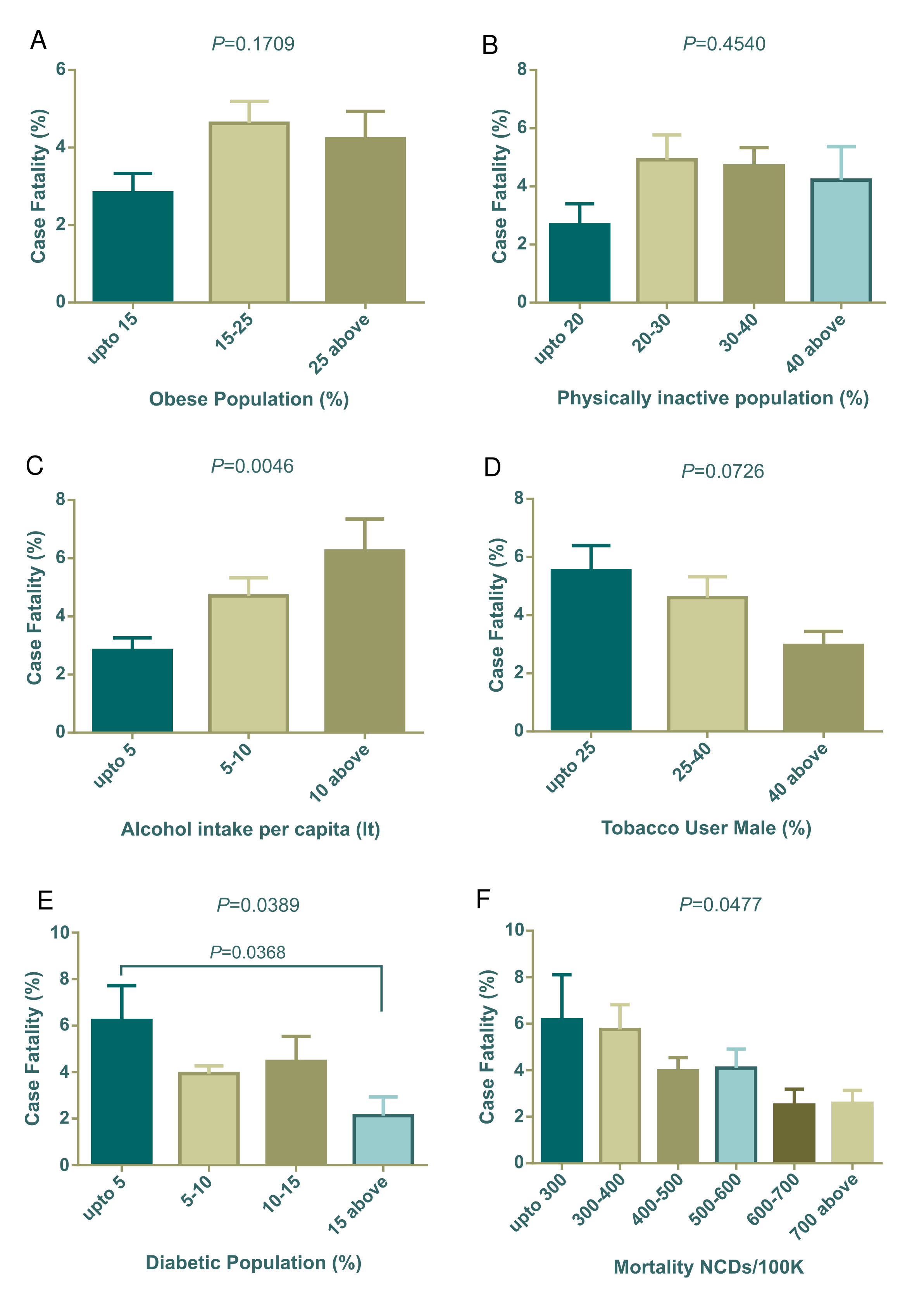

Supplement: Supplementary file 4 [file Figure_4.TIF]

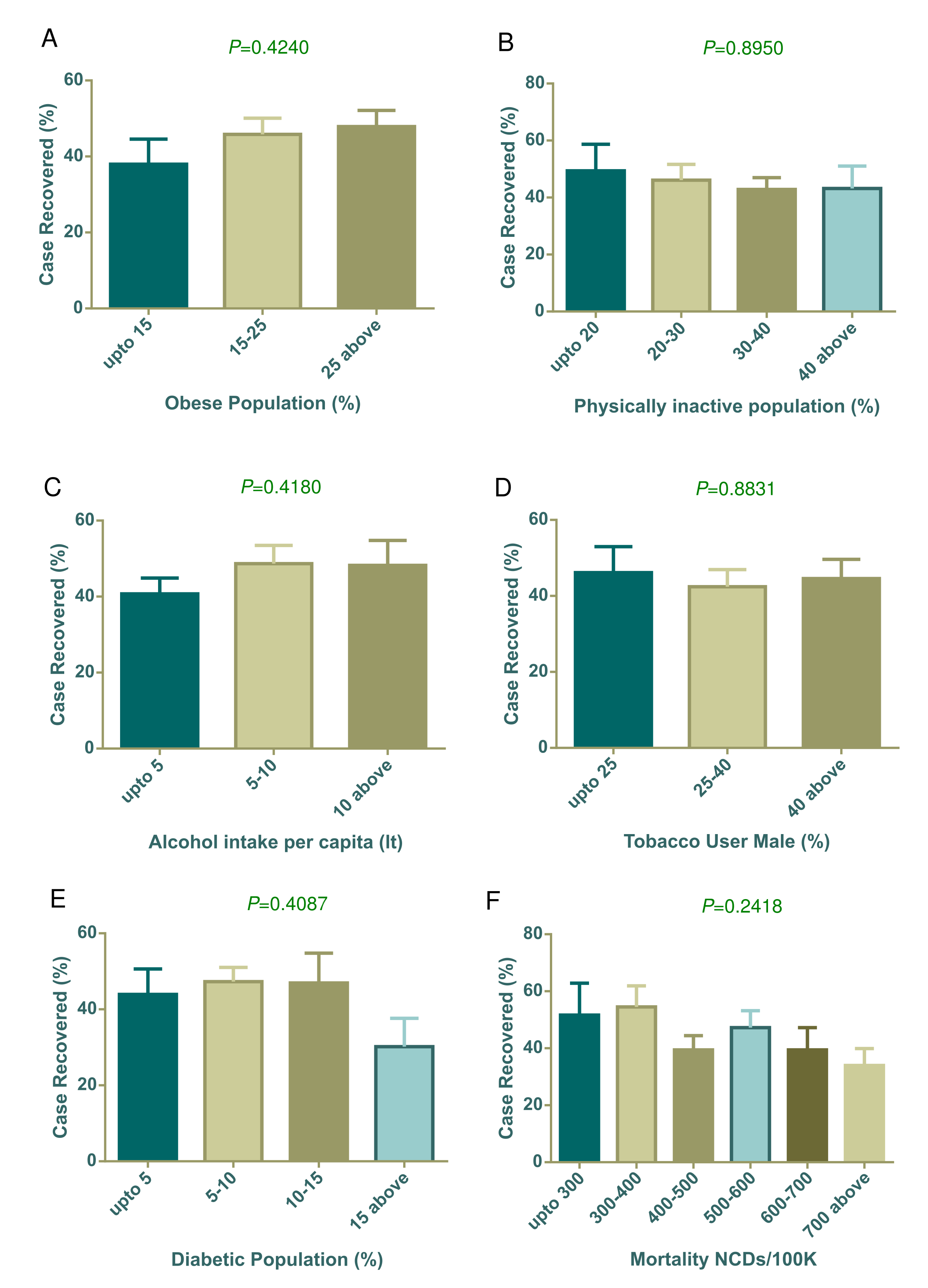

Supplement: Supplementary file 5 [file Figure_5.TIF]

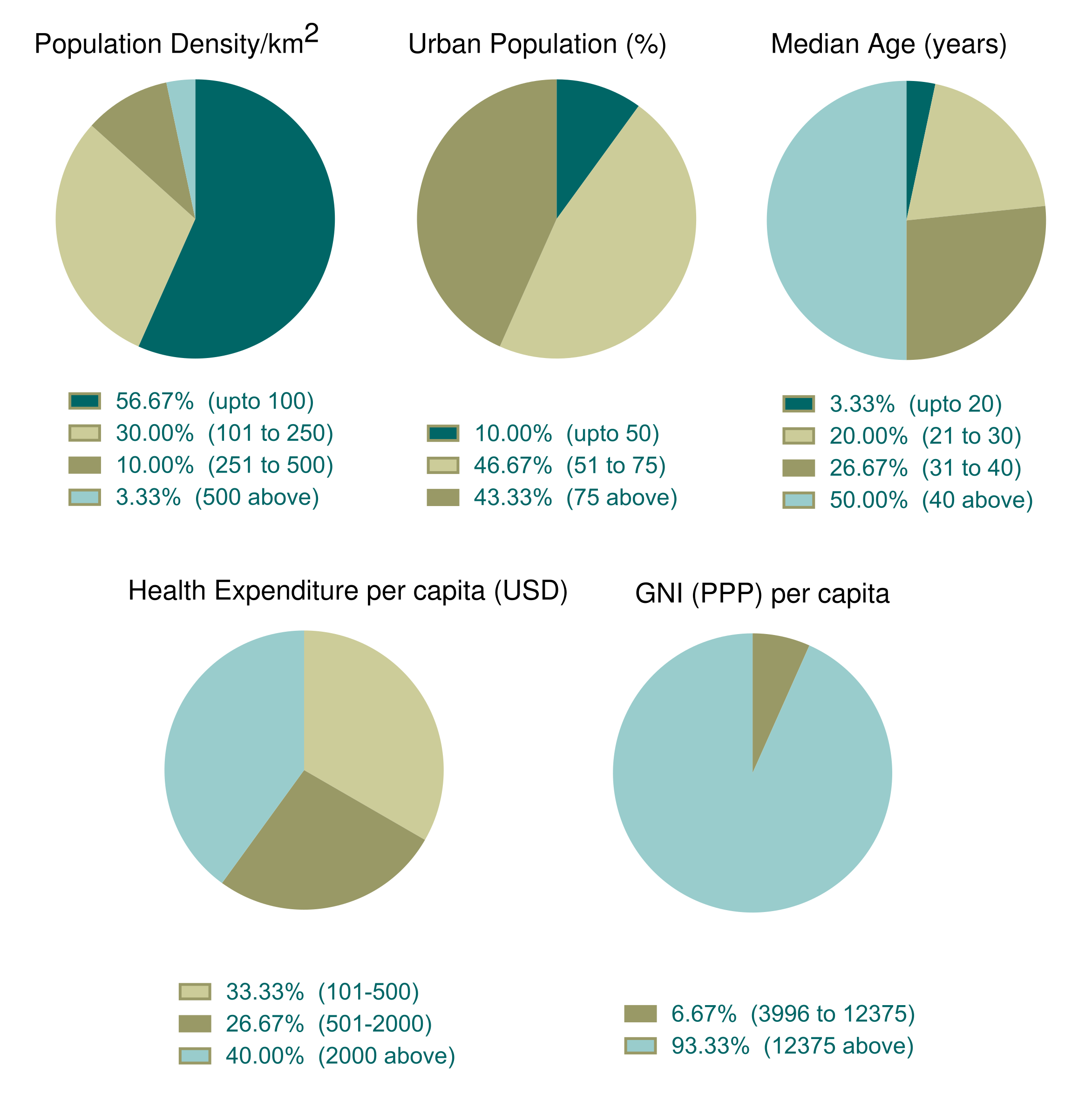

Supplement: Supplementary file 6 [file Figure_6.TIF]

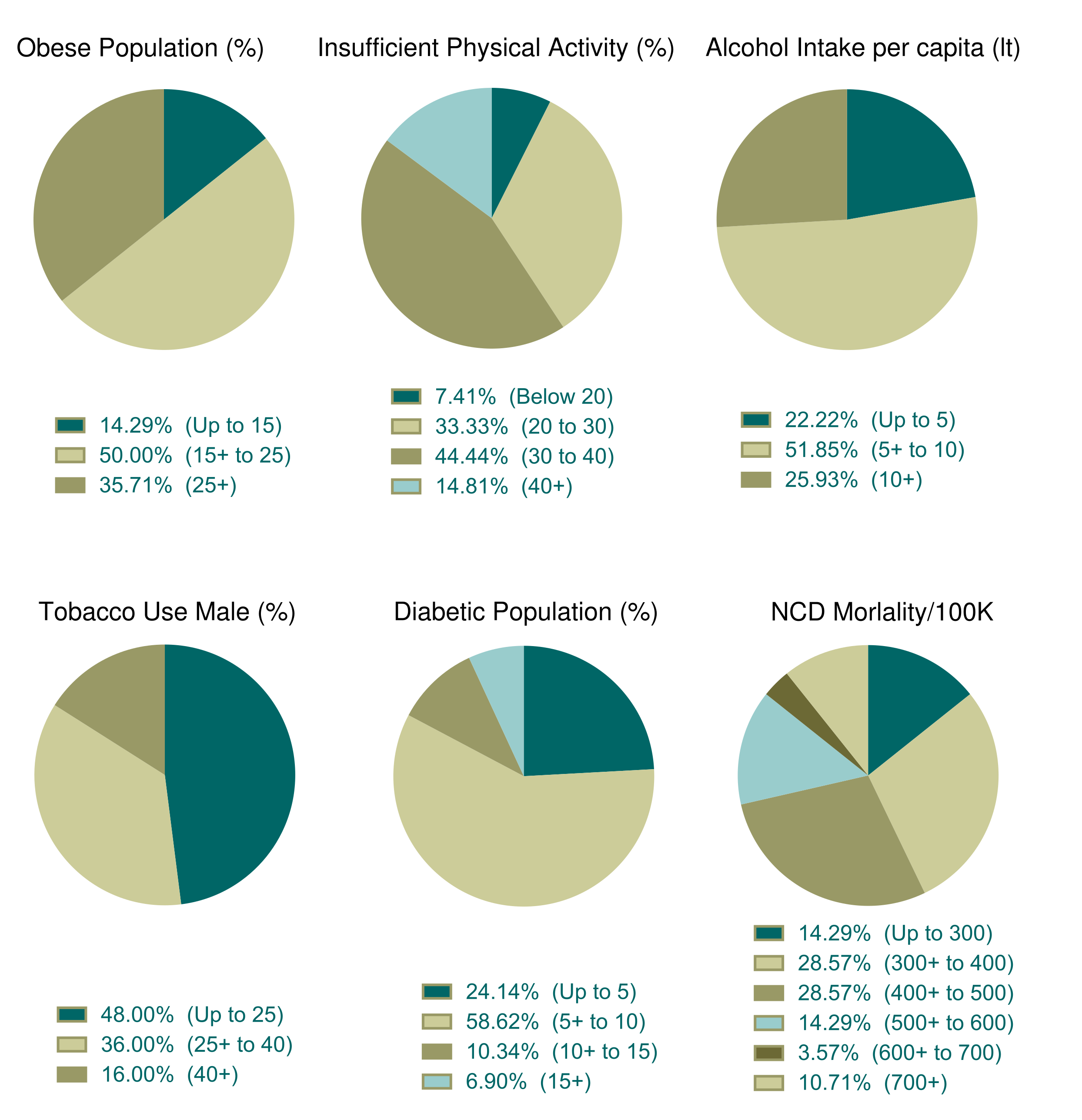

Supplement: Supplementary file 7 [file Figure_7.TIF]
