# Supplementary material for: Lifestyle Acquired Immunity, Decentralized Intelligent Infrastructures, and Revised Healthcare Expenditures May Limit Pandemic Catastrophe: A Lesson From COVID-19
Source: Front Public Health. 2020 Nov 5;8:566114. doi: 10.3389/fpubh.2020.566114 (PMC7674625; doi:10.3389/fpubh.2020.566114)
Supplement: Supplementary file 9 [file Table_2.DOCX]

Lifestyle acquired immunity, decentralized intelligent infrastructures and revised healthcare expenditures may limit pandemic catastrophe: a lesson from COVID-19

Asif Ahmed^1*^, Tasnima Haque^2^ and Mohammad Mahmudur Rahman^3^

^1^Biotechnology and Genetic Engineering Discipline, Khulna University, Khulna 9208, Bangladesh

^2^BIHS General Hospital, Bangladesh

^3^Department of Medical Biotechnology, Bangladesh University of Health Sciences, Bangladesh

*** Correspondence:**Asif Ahmed
asif@bge.ku.ac.bd

**Supplementary**

**Table 1: Association between Prevalence of Diabetes and Median age of studied countries**

|  | Median age, years | | | |
| --- | --- | --- | --- | --- |
| Prevalence of Diabetes | Up to 20 | 20+ to 30 | 30+ to 40 | 40+ |
| Up to 5% | 3 | 3 | 2 | 10 |
| 5%+ to 10% | 2 | 11 | 20 | 23 |
| 10%+ to 15% | 0 | 4 | 3 | 1 |
| 15%+ | 1 | 3 | 4 | 0 |

**Table 2: Association between Prevalence of Diabetes and per capita GNI (PPP) of studied countries**

|  | Per capita GNI (PPP) in USD | | | |
| --- | --- | --- | --- | --- |
| Prevalence of Diabetes | Up to 1026 | 1026 to 3995 | 3995 to 12375 | 12375+ |
| Up to 5% | 0 | 2 | 3 | 13 |
| 5%+ to 10% | 0 | 2 | 12 | 42 |
| 10%+ to 15% | 0 | 0 | 1 | 7 |
| 15%+ | 0 | 0 | 3 | 5 |

**Table 3: Association between NCD Mortalities per 100K and Median age of studied countries**

|  | Median age, years | | | |
| --- | --- | --- | --- | --- |
| NCD Mortality per 100K | Up to 20 | 20+ to 30 | 30+ to 40 | 40+ |
| Up to 300 | 0 | 0 | 2 | 5 |
| 300+ to 400 | 0 | 0 | 8 | 12 |
| 400+ to 500 | 0 | 9 | 8 | 5 |
| 500+ to 600 | 0 | 4 | 5 | 5 |
| 600+ to 700 | 2 | 2 | 2 | 5 |
| 700+_ | 4 | 5 | 3 | 0 |

**Table 4: Association between NCD Mortalities per 100K and per capita GNI (PPP) of studied countries**

|  | GNI | | | |
| --- | --- | --- | --- | --- |
| NCD Mortality per 100K | Up to 1026 | 1026 to 3995 | 3995 to 12375 | 12375+ |
| Up to 300 | 0 | 0 | 0 | 7 |
| 300+ to 400 | 0 | 0 | 0 | 20 |
| 400+ to 500 | 0 | 0 | 3 | 19 |
| 500+ to 600 | 0 | 0 | 4 | 10 |
| 600+ to 700 | 0 | 2 | 5 | 4 |
| 700+_ | 0 | 2 | 7 | 3 |
